# Determinants of Quality of Life in Ageing Populations: Results from a Cross-Sectional Study in Finland, Poland and Spain

**DOI:** 10.1371/journal.pone.0159293

**Published:** 2016-07-19

**Authors:** Alberto Raggi, Barbara Corso, Nadia Minicuci, Rui Quintas, Davide Sattin, Laura De Torres, Somnath Chatterji, Giovanni Battista Frisoni, Josep Maria Haro, Seppo Koskinen, Andrea Martinuzzi, Marta Miret, Beata Tobiasz-Adamczyk, Matilde Leonardi

**Affiliations:** 1 Neurological Institute C. Besta IRCCS Foundation, Neurology, Public Health and Disability Unit, Milan, Italy; 2 National Council Research, Neuroscience Institute, Padova, Italy; 3 World Health Organization, Information, Evidence and Research Unit, Geneva, Switzerland; 4 IRCCS Istituto Centro San Giovanni di Dio-Fatebenefratelli, Laboratory of Alzheimer's Neuroimaging and Epidemiology, Brescia, Italy; 5 Memory Clinic and LANVIE, University Hospitals and University of Geneva, Geneva, Switzerland; 6 Parc Sanitari Sant Joan de Déu, University of Barcelona, CIBERSAM, Barcelona, Spain; 7 National Institute for Health and Welfare, Population Health Research Unit, Helsinki, Finland; 8 E. Medea Scientific Institute, Conegliano-Pieve di Soligo Research Centre, Conegliano Veneto, Italy; 9 Department of Psychiatry, Universidad Autónoma de Madrid, Madrid, Spain; 10 Instituto de Salud Carlos III, Centro de Investigación Biomédica en Red de Salud Mental, CIBERSAM, Spain; 11 Department of Psychiatry, Hospital Universitario de La Princesa, Instituto de Investigación Sanitaria Princesa (IP), Madrid, Spain; 12 Department of Medical Sociology, Chair of Epidemiology and Preventive Medicine, Jagiellonian University Medical College, Krakow, Poland; CSIR-Central Drug Research Institute, INDIA

## Abstract

**Purpose:**

To comprehensively identify the determinants of quality of life (QoL) in a population study sample of persons aged 18–50 and 50+.

**Methods:**

In this observational, cross-sectional study, QoL was measured with the WHOQOL-AGE, a brief instrument designed to measure QoL in older adults. Eight hierarchical regression models were performed to identify determinants of QoL. Variables were entered in the following order: Sociodemographic; Health Habits; Chronic Conditions; Health State description; Vision and Hearing; Social Networks; Built Environment. In the final model, significant variables were retained. The final model was re-run using data from the three countries separately.

**Results:**

Complete data were available for 5639 participants, mean age 46.3 (SD 18.4). The final model accounted for 45% of QoL variation and the most relevant contribution was given by sociodemographic data (particularly age, education level and living in Finland: 17.9% explained QoL variation), chronic conditions (particularly depression: 4.6%) and a wide and rich social network (4.6%). Other determinants were presence of disabling pain, learning difficulties and visual problems, and living in usable house that is perceived as non-risky. Some variables were specifically associated to QoL in single countries: age in Poland, alcohol consumption in Spain, angina in Finland, depression in Spain, and self-reported sadness both in Finland and Poland, but not in Spain. Other were commonly associated to QoL: smoking status, bodily aches, being emotionally affected by health problems, good social network and home characteristics.

**Conclusions:**

Our results highlight the importance of modifiable determinants of QoL, and provide public health indications that could support concrete actions at country level. In particular, smoking cessation, increasing the level of physical activity, improving social network ties and applying universal design approach to houses and environmental infrastructures could potentially increase QoL of ageing population.

## Introduction

European population is undergoing an unprecedented ageing process, which is taking place as a joint effect of increased life expectancy and reduced fertility: the percentage of persons aged 60+ increased from 9.2% in 1990 to 11.7% in 2013 and is projected to reach 21.1% by 2050 [1]. Increased life expectancy leads to increased prevalence of non-communicable diseases and to a relevant increase on the burden associated to these conditions: in fact, the increase in years lived with disability in the last two decades was 55.4% for non-communicable diseases, 7.6% for communicable disease and 0.3% for injuries [2].

In such a context, healthy ageing is becoming an important pillar of research and an objective for policy-makers. Healthy ageing is defined as “the process of developing and maintaining the functional ability that enables well-being in older age” [3]. Healthy ageing is expected to impact on quality of life (QoL): thus, understanding the features of QoL, and of its determinants, in a healthy ageing perspective is of primary relevance. Studies addressing determinants of QoL generally focus on a limited number of domains, such as the presence of multi-morbidities [4,5], visual impairment [6] and obesity [7], behavioral issues, such as higher levels of alcohol use [8,9], smoking [10,11] or active lifestyle [9,12]. In addition to this, social factors have also been shown to influence QoL in the ageing process: examples of this include social and family relationships [13,14] and socioeconomic status [15,16].

Research addressing the impact of a larger number of factors on QoL is, however, almost lacking. In particular, little research has been devoted to the analysis of the impact of built environment (i.e. the part of the environment which results as an effect of human activity) on QoL [17], while some indications of its impact on the health state have been found: living in walkable environments are associated with increased physical activity, lower prevalence of overweight, lower depression and less reported alcohol abuse [18]; better-quality built environment is associated with lower prevalence of psychiatric symptoms [19]; worse-quality built environment is associated with poorer self-rated health [20]. The aim of this paper is to comprehensively identify the determinants of QoL in a large population study sample of persons aged 18–50 and 50+ that were enrolled on occasion of the COURAGE in Europe Project (Collaborative Research on Ageing in Europe). We included a wide set of candidate determinants, such as demographic data, chronic conditions, health and health habits information, as well as social networks and built environment variables. We relied on the definition of QoL endorsed by the WHO’s WHOQOL group: QoL as the individuals’ perception of their position in life in the context of the culture and value systems in which they live and in relation to their goals, expectations, standards and concerns [21].

## Methods

### Study design, procedure and sample

COURAGE in Europe is an observational, cross-sectional study of the general community dwelling adult population reached though face-to-face household interviews that were conducted between May 2011 and March 2012 in Finland, Poland and Spain using a computer-assisted personal interviewing system. The instruments used were translated from English into Finnish, Polish, and Spanish following the World Health Organization translation guidelines for assessment instruments [22]. Quality assurance procedures were implemented during fieldwork [23]. The present study was approved by the ethical committee of Neurological Institute Carlo Besta, Milan, Italy, project coordinator; the Ethics Review Committee, National Public Health Institute, Helsinki, Finland; the Bioethical Committee, Jagiellonian University, Krakow, Poland; Ethics Review Committee, Parc Sanitari Sant Joan de Déu, Barcelona, Spain; and Ethics Review Committee, La Princesa University Hospital, Madrid, Spain. Written informed consent from each participant was also obtained. Please see S1 File to access available data.

The three countries were selected to give a broad representation across different geographical European regions. A multi-stage clustered design was used to obtain nationally representative samples. The whole sample comprised 10,800 respondents: 1976 from Finland (response rate 53.5%), 4071 from Poland (response rate 66.5%), and 4753 from Spain (response rate 69.9%) [24].

### Measures

#### Quality of Life

QoL was measured with the WHOQOL-AGE, a 13-items instrument that was based on other versions of the WHOQOL. The development and validation is fully described elsewhere [25]: the WHOQOL-AGE is designed and validated to measure QoL in older adults. and provides a single score that ranges on a 0–100 scale, with higher scores indicating higher QoL.

#### Socio-demographic information

Socio-demographic information included age, educational attainment divided into three categories (None, Primary or Secondary school, High school or higher), marital status categorized into Married or cohabiting, Never married, Separated or Divorced, and Widowed and location, grouped into urban or rural according to country-specific definitions.

#### Health Habits

Health Habits included in the analysis were: smoking status, alcohol consumption and physical activity, plus body mass index (BMI) and waist circumference (WC).

Height and weight were measured with the use of a stadiometer and a routinely calibrated electronic weighting scale respectively. BMI was calculated by dividing measured weight (in kilograms) by squared height (in meters), i.e. kg/m^2^. BMI was categorized as follows: underweight <18.4; normal 18.5–24.9; overweight 25.0–29.9; obese ≥30.

According to the WHO standards, WC was categorized into low risk (WC in the range 40–102 for males and 54–88 for females) and high risk (WC in the range 102.1–152 for males and 88.1–156 for females) [26].

Respondents were classified as current smokers, past smokers or those who had never smoked Users of smokeless tobacco were excluded from the analysis.

With regard to alcohol consumption, questions addressed individual consumption patterns, including frequency and quantity of alcohol use. Responders were grouped into four groups [27]:

1. lifetime abstainers or occasional drinkers (i.e. those who had never consumed an alcoholic beverage or had not consumed alcohol in the last 30 days);
2. non-heavy drinkers (i.e. social drinkers who consumed alcohol in the last 30 days but were not heavy drinkers);
3. infrequent heavy drinkers (i.e. binge drinkers who consumed alcohol on 1–2 days in the past week with 5 or more standard drinks for men and 4 or more standard drinks for women);
4. frequent heavy drinkers (those who consumed alcohol on 3 or more days per week with 5 or more standard drinks for men and 4 or more standard drinks for women).

Questions about the type and level of physical activity that the respondent undertakes were based on the second version of the Global Physical Activity Questionnaire (GPAQ v2) [28]. The GPAQ v2 differentiates between work and leisure, and recreational and sport-related activities, and records the frequency (number of days) and duration (minutes or hours) of each activity undertaken in the preceding 7 days. Using conventional cut-off points the following levels of physical activity were created [29]:

1. high physical activity (vigorous-intensity activity on at least 3 days achieving a minimum of at least 1500 Metabolic Equivalent to Task (MET)-minutes per week or—7 or more days of any combination of walking, moderate or vigorous intensity activities achieving a minimum of at least 3000 MET-minutes per week);
2. moderate physical activity (3 or more days of vigorous-intensity activity of at least 20 minutes per day; 5 or more days of moderate-intensity activity or walking of at least 30 minutes per day; or 5 or more days of any combination of walking, moderate or vigorous intensity activities achieving a minimum of at least 600 MET-minutes per week);
3. low physical activity (a person not meeting any of the above mentioned criteria).

#### Assessment of Chronic Conditions

Prevalence estimates of chronic conditions were based on self-report by respondents through the question, “Has a health care professional ever told you, you have…?" for the following eight conditions: Arthritis, Stroke, Angina, Diabetes, Lung disease, Asthma, Depression, and Hypertension.

#### Health State description

Respondents were asked a series of questions on symptoms or difficulties, including pain, learning, concentration, sleep, feeling tired, sad or depressed, feelings of worry or anxiety, being emotionally affected by one’s own health state, and a general rating of how much these problems/difficulties interfere with respondents’ lives. All items were rated on a 5-point scale (no problems, mild, moderate, severe, extreme/complete problems). With the exception of the question on pain, that kept was on a 5-point scale (no pain, pain but no problems, pain and mild problems, pain and moderate problems, pain and severe problems), responses referring severe and extreme/complete problems were merged as the option “extreme/complete” was reported by less than 1% of respondents.

#### Vision and Hearing

These constructs were addressed with four questions, referred to the previous 30 days: a) Distant vision, with the response to the question “how much difficulty did you have in seeing and recognizing an object or a person you know across the road (from a distance of about 20 meters)?”; b) Near vision, with the response to the question “how much difficulty did you have in seeing and recognizing an object at arm's length (for example, reading)?”; c) Near hearing, with the response to the question “how much difficulty did you have in: hearing someone talking on the other side of the room in a normal voice (even with your hearing aid on if you use one)?”; d) Problems with conversation hearing, with the response to the question “how much difficulty did you have in: hearing what is said in a conversation between several people (even with your hearing aid on if you use one)?”. All items were rated on a 4-point scale (no problems, mild, moderate, extreme/complete problems).

#### Social Networks

A synthetic social networks index (SNI), fully described elsewhere [30] was used to evaluate the impact of social networks. Briefly social network was defined as a multidimensional set of independent networks involving the relations with spouse, parents, other relatives (children, grandchildren and others), neighbours, friends and co-workers. For each of them, structural and functional aspects were taken into account, namely, the size of specific networks, the ties (close relations), help (general social support) and the frequency of face-to-face contacts. The SNI ranged from 0 to 100, with higher score indicating better social networks.

#### Built Environment

Courage Built Environment self-reported questionnaire (CBE-SR) was fully described elsewhere [31]. It comprises 19 items grouped into four indexes: “Usability of the neighbourhood environment”, “Hindrance of walkable environment”, “Easiness of use of public buildings, places and facilities”, “Usability of the living place”. For each of the four scales, scores range between 0 and 100: higher scores address, respectively, a neighborhood environment perceived as more usable, walkable environment perceived as more hindering, public buildings, places and facilities perceived as more easy to use, and living place perceived as less risky and more usable.

### Statistical analyses

Descriptive statistics are presented overall and by country as percentage or mean values (± standard deviation) as appropriate. Weighted data have been used to account for sampling design, and post-stratification corrections to the weights have been used to adjust for population distribution and for non-responses.

Eight multivariable hierarchical regression models were performed in order to identify possible determinant of QoL. The hierarchical models were implemented as follows:

- Model 1: Sociodemographic variables;
- Model 2: significant predictors from Model 1 and Health Habits;
- Model 3: significant predictors from Model 2 and Chronic Conditions;
- Model 4: significant predictors from Model 3 and Health State description;
- Model 5: significant predictors from Model 4 and Vision and Hearing;
- Model 6: significant predictors from Model 5 and SNI;
- Model 7: significant predictors from Model 6 and CBE;
- Model 8: the final model retained only the significant variables of Model 7.

The final model was replicated selecting responders country by country from the three countries so to address similarities and differences across Finland, Poland and Spain.

The SAS’ surveyreg procedure was used for the construction of the models to take into account the nature of the complex sample design, including the individual weights, cluster and strata. Analysis of residuals was performed to examine models’ goodness of fit and adherence to regression assumptions. Multicollinearity was checked using the tolerance and the Variance Inflation Factor; variables with tolerance < 0.4 and Variance Inflation Factor >2.5 were discarded from the analysis. Specifically, the variables “Feel tired”, “Problems with worry or anxiety”, “Difficulties interfere with life”, “Hearing problem in conversation with several people” were discarded from Models 4 and 5 due to their multicollinearity with “Difficulty in sleep”, “Feel sad, low or depressed”, “Emotionally affect by health problems”, “Near hearing”, respectively.

The significant level was set at 0.05. All statistical analyses were performed using SAS, version 9.4.

## Results

The final sample with complete information across all variables, described in Table 1, comprised 5639 participants, mean age 46.3 (SD 18.4). Most of respondents were from urban contexts and the sample was balanced for gender. Responders from Poland reported lower QoL scores. Please refer to S1 Table for the complete distribution of variables by country.

**Table 1:** Distribution of variables by country.

	Totaln = 5639	Finlandn = 520	Polandn = 2863	Spainn = 2256
***Socio/ Demographic***				
Age in years (mean±sd)	46.3±18.4	57.0±16.1	44.6±18.3	46.1±18.3
Urban Location	80.6	90.3	72.3	89.4
Female gender	51.2	50.8	52.1	50.2
Education				
Secondary or less	41.7	27.2	37.8	50.0
High school & Higher education	58.3	72.8	62.2	50.0
Marital status				
Never married	24.6	19.1	23.1	27.9
Married/cohabiting	60.3	59.2	63.2	56.8
Separate/Divorced/Widowed	15.1	21.7	13.7	15.3
***Health habits***				
BMI				
Overweight	35.4	39.5	33.3	37.2
Obese	22.9	23.6	23.6	21.9
Waist risk–high	33.0	40.0	29.3	36.2
Smoking status				
Never smoked/Ex-smoker	68.6	80.6	68.8	65.6
Current smoker	31.4	19.4	31.2	34.4
Alcohol consumption				
Not heavy drinker	54.4	53.4	56.4	51.8
Heavy drinker (frequent & infrequent)	7.9	17.0	8.2	5.6
Physical activity				
Moderate	27.0	34.6	17.3	38.1
High	50.8	49.1	61.0	37.7
***Chronic conditions***				
Arthritis	15.5	37.5	14.8	11.4
Stroke	2.0	3.4	2.0	1.7
Angina	4.4	8.1	5.0	2.9
Diabetes	6.5	8.8	6.5	6.1
Lung disease	4.4	3.4	4.8	4.3
Asthma	6.4	9.1	5.5	7.0
Depression	11.3	15.1	7.8	15.0
Hypertension	23.1	32.2	25.8	17.6
***Health state***				
Bodily aches or pains				
Pain and mild/moderate difficulty	29.7	18.1	36.2	23.8
Pain and severe/extreme difficulty	6.6	1.6	8.2	5.6
Difficulty in learning a new task				
Mild/Moderate	12.9	23.5	15.0	7.9
Severe/Extreme	1.9	1.9	2.5	1.1
Difficulty in concentrating				
Mild/Moderate	8.6	7.8	9.8	7.3
Severe/Extreme	1.0	0.7	1.1	0.9
Difficulty in sleep				
Mild/Moderate	28.1	40.7	29.9	22.9
Severe/Extreme	7.1	8.7	7.8	5.9
Feel tired				
Mild/Moderate	31.0	51.8	35.8	19.8
Severe/Extreme	4.3	4.1	5.0	3.5
Feel sad, low or depressed				
Mild/Moderate	34.8	26.5	44.3	24.2
Severe/Extreme	4.4	1.5	5.2	3.9
Problems with worry or anxiety				
Mild/Moderate	36.3	26.2	46.2	25.5
Severe/Extreme	4.4	1.6	5.3	3.9
Emotionally affect by health problems				
Mild/Moderate	26.7	26.9	32.3	19.4
Severe/Extreme	4.8	3.3	6.2	3.3
Difficulties interfere with your life				
Mild/Moderate	28.4	36.4	34.5	19.3
Severe/Extreme	3.8	2.7	4.6	3.1
***Vision/ Hearing***				
Distant vision				
Mild/Moderate	13.0	9.8	15.1	10.1
Severe/Extreme	1.4	1.4	2.0	0.6
Near vision				
Mild/Moderate	13.5	10.1	15.9	11.1
Severe/Extreme	1.1	0.8	1.7	0.4
Near hearing				
Mild/Moderate	11.2	22.4	10.6	9.5
Severe/Extreme	1.4	1.0	1.6	1.3
Hearing problem in conversation with several people				
Mild/Moderate	9.9	12.0	11.0	8.1
Severe/Extreme	1.4	0.4	2.1	0.7
***SN index***				
Social Network Score (mean±sd)	70.0±12.8	63.0±9.9	67.5±12.3	74.8±12.5
***BE Indexes***				
Reachability and usability of the neighbourhood environment (mean±sd)	70.1±25.1	61.5±20.3	69.4±23.4	72.9±27.9
Hinderance of walkable environment (mean±sd)	29.3±25.5	14.2±14.9	35.2±24.8	24.8±26.4
Open-to-public buildings, places and facilities (mean±sd)	73.1±24.3	73.2±18.6	65.8±23.8	82.8±22.8
Usability of the living place/home (mean±sd)	76.8±23.2	80.6±19.5	71.7±23.1	82.7±22.7
***Quality of Life***				
Quality of life Score (mean±sd)	72.4±14.6	78.1±10.6	69.9±14.6	74.4±14.9

*Notes*. All values are percentage by country, except where reported differently.

Table 2 reports the results of the hierarchical regression analysis. The final model accounted for 45% of the variation (49.4% in Finland, 47.7% in Poland and 40.2% in Spain) and the most relevant contribution, in terms of explained QoL variation, was given by sociodemographic data (Model 1: 17.9% explained QoL variation), chronic conditions (Model 3: 4.6% additional explained QoL variation) and SNI (Model 6: 4.6% additional explained QoL variation). Please refer to S2 Table for the full hierarchical regression models to predict quality of life.

**Table 2:** Hierarchical regression models to predict quality of life.

**Domains and variables**	Total (n = 5639; R^2^:0.450)	Finland(n = 520; R^2^:0.494)	Poland(n = 2863; R^2^:0.477)	Spain(n = 2256; R^2^:0.402)
***Model 1*: *Socio/ Demographic (R***^***2***^ ***= 0*.*1793)***				
Country (ref. Poland)				
Finland	7.40***	–	–	–
Spain	0.34	–	–	–
Age (years)	-0.09***	-0.04	-0.17***	-0.004
Education (ref. Higher education)				
None	-2.79	No data	-3.75	-5.35***
Primary + Secondary	-2.53***	-1.20	-2.63*	-3.36***
***Model 2*: *Health habits (R***^***2***^ ***= 0*.*2118)***				
Smoking status (ref. Never smoked)				
Ex-smoker	-0.49	-1.11	-0.71	-0.12
Current Smoker	-2.80***	-4.83***	-2.32*	-2.88**
Alcohol consumption (ref. Abstainer)				
Not heavy drinker	1.65**	-1.90	1.43	2.26**
Infrequent heavy drinker	2.46*	0.05	2.23	3.56
Frequent heavy drinker	3.66	1.35	4.05	3.79
Physical activity (ref. High)				
Low	-1.98**	-2.31	-2.22*	-1.51
Moderate	0.21	-1.93*	0.46	0.47
***Model 3*: *Chronic conditions (R***^***2***^ ***= 0*.*2579)***				
Angina (ref. No)	-2.28*	-2.84**	-1.99	-2.24
Depression (ref. No)	-3.18***	0.79	-2.55	-4.33***
***Model 4*: *Health state (R***^***2***^ ***= 0*.*3725)***				
Bodily aches or pains (ref. No pain)				
Pain but no difficulty	-1.47	-2.41**	0.63	-3.12*
Pain and mild difficulty	-2.70**	-3.04*	-3.18**	-1.77
Pain and moderate difficulty	-3.36***	-4.02*	-1.98	-4.93***
Pain and severe/extreme difficulty	-5.53***	-10.16***	-4.11**	-6.09**
Difficulty in learning a new task (ref. None)				
Mild	-0.34	-1.94	0.83	-2.91
Moderate	-4.21***	-2.48	-2.87*	-6.94**
Severe/Extreme	-5.27***	-2.80	-5.13**	-3.82
Feel sad, low or depressed (ref. None)				
Mild	-2.81***	-2.40**	-3.26**	-2.25*
Moderate	-3.53***	-7.89**	-3.96***	-2.93
Severe/Extreme	-7.07***	-10.29***	-8.43***	-5.36
Emotionally affect by health problems (ref. No)				
Mild	-2.66**	-4.24***	-2.48	-2.44*
Moderate	-4.87***	-3.28*	-5.42***	-4.28**
Severe/Extreme	-4.33*	-5.10*	-3.59*	-5.61
***Model 5*: *Vision/ Hearing (R***^***2***^ ***= 0*.*3757)***				
Distant vision (ref. None)				
Mild	-1.20	-0.48	-0.85	-2.03
Moderate	-1.77	-0.30	-2.85*	1.16
Severe/Extreme	-4.22***	-6.04*	-3.67*	0.38
***Model 6*: *Social Network index (R***^***2***^ ***= 0*.*4213)***				
Social Network Score	0.24***	0.32***	0.22***	0.24***
***Model 7*: *Built Environment Indexes (R***^***2***^ ***= 0*.*4539)*.**				
Usability of the living place	0.11***	0.10***	0.10***	0.13***

*Note*:

*P≤0.05

**P≤0.01

***P≤0.001.

The following predictors had the most significant direct association with higher QoL scores: living in Finland contrasted to living in Poland, having a good social network and living in a house that is perceived as usable and with low risk of accidents. The following predictors had the most significant inverse association with higher QoL scores: age, lower education levels (primary or secondary) contrasted to higher education, being an active smoker contrasted to being a long-life never smoker, being depressed, self-reporting pain determining moderate to extreme difficulties compared to no pain, moderate to extreme learning difficulties compared to no difficulties, feelings of sadness of any extent, self-reporting to be moderately emotionally affected by one’s own health condition contrasted to not being affected and, finally, self-reporting severe/extreme distant vision problems.

Some variables were basically consistently associated to QoL in the three countries: these include variables with an inverse association with QoL such as smoking status, bodily aches and pain, being emotionally affected by health problems, as well as variables directly associated, such as good social network and living in a house that is perceived as usable and with low risk of accidents. Other variables, on the contrary, had a specific role across countries: age was significantly associated to QoL only in Poland, alcohol consumption only in Spain, angina was significantly associated only in Finland and depression only in Spain, self-reported feelings of sadness were significantly associated both in Finland and Poland, but not in Spain.

## Discussion

With this study, we reported on the determinants of QoL using data referred to 5639 persons from Finland, Poland and Spain. Our results show that the most important factors were sociodemographic variables (age, education level and living in Finland), negative health habits (smoking status and physical inactivity), presence of chronic conditions, particularly depression, presence of disabling pain, of learning difficulties and of visual problems, a wide and rich social network, and living in usable house that is perceived as non-risky.

To our knowledge, this is one of the few studies addressing a wide range of determinants of QoL from a population study. Thompson and colleagues [32] addressed the effect of several variables over composite measures of physical and mental QoL in U.S. adults aged 50 and over. They found that medical care costs, smoking and leisure-time physical activity (i.e. practicing physical activities in addition to work-related ones) had large effects on both mental and physical aspects of QoL, particularly among those with functional limitations. Layte and colleagues [33] addressed the determinants of QoL in an Irish sample of subjects aged 50 and over: they found that mental health, social participation and physical health explain more than 60% of the variation of QoL, with mental health issues having the strongest independent power. The results of our study are in line with those herein described, but expand their value by adding some data, such as education and the characteristics of respondents’ homes, by the fact that we controlled for a large number of variables and by the fact that we enrolled subjects from 18 years on.

Our results can be broadly divided in two areas: the determinants of QoL that are not, or little, likely to be modified if not in the long run, and those that might be modified with short-medium term interventions. In the first group, variables such as age and education level–that however might change among the younger respondents–and visual problems are included; the second group is composed of health habits such as smoking, alcohol consumption, physical inactivity, presence of pain as well as by less frequently addressed variables like social network and the features of the built environment.

The relation between individual QoL and age is controversial. In some studies, age was found among the predictors of decreased QoL, most likely as a result of the effect of chronic conditions in older individuals [5,34], while such a relationship was not confirmed in other studies [4,35]. Our results show that age determined worse QoL when controlling for a wide set of variables, and we did not find a relevant impact of comorbidities like in previous studies: it is to be noted that only angina and depression remained significant in our final model. Severe distant vision problems had a strong negative impact on QoL, which basically confirmed previous research findings [36,37]. Similarly to what shown in other studies, we also found that distant vision, and not near vision, impact on QoL [38] and that positive relationships have a mitigating role [39]; on the contrary, we did not find any effect of hearing impairment that was previously shown to impact on QoL [40].

Education has been sometimes used as a way to address socioeconomic status (SES) inequalities connected to health and QoL: our findings show that lower educational levels are associated to diminished QoL compared to having high school or academic degrees. Similar results were found among participants to the American Health and Retirement Study and to the English Longitudinal Study of Ageing [41], as well as among community-dwelling elderly individuals (aged 65+) from Switzerland [42]. As previously reported [16] people with low SES seem to face a double burden: first, increased levels of health impairments and, second, lower QoL once health is impaired. Our data show that lower education levels, presence of health state problems–specifically connected to pain and emotional difficulties–and presence of chronic conditions such as angina and depression determine lower QoL. Angina and depressive symptoms were already found to be associated with and impaired QoL [43,44], and pain is frequently reported in association with depression or low mood: a recent study showed that these two symptoms have a distinct impact over reduced QoL [45], while other hypothesized that they show a bidirectional interaction and are correlated [46,47]: our results seem to be consistent with the hypothesis of a distinct impact of pain and depressive symptoms on reduced QoL. Our data did not address the nature of pain, which can therefore have any origin and can be associated to prevalent conditions, e.g. musculoskeletal disease like neck or back pain, or neurological conditions such as headache disorders. Presence of musculoskeletal pain was in fact associated to lower QoL, with higher pain severity and multiple locations being associated to worse QoL [48–50]. Pain from headache disorders were shown to influence QoL through both pain intensity and frequency of headaches, with more frequent headaches determining worse QoL [51,52].

As reported in a recent review, a negative relationship between smoking and QoL exists and the magnitude of association is consistent with the amount of cigarettes smoked [53]. Smoking cessation was found to significantly improve QoL, which was found to be better between 2 and 5 years after quitting [11].Our data seem to support these findings: contrasted to never smoking, smoking status determined worse QoL, while no predictive power was found for previous smoking. However, we did not include information on the duration of non-smoking period, therefore this has to be taken just as an hypothesis. Smoking has relevant implications, not only connected to QoL, but also to general health and preventive policies. Compared to non-smokers, smokers were more likely to have problems with mobility, self-care and daily activities, and were also more likely to report pain, anxiety and depression [11]. Smoking is a risk factor that tends to present with other factors, such as alcohol consumption and physical inactivity [54,55], which is partly consistent with our results. Low levels of physical activity were in fact found to be associated to reduced QoL in a large number of studies [56,57], as well as to obesity or overweight [58,59]. In contrast with these results, our data did not support the association between obesity, increased WC and QoL, despite a more than half of our sample was obese or overweight and one-third had high-risk WC. The issue of alcohol consumption deserves a separate comment, since a moderate alcohol consumption was associated to better QoL compared to being abstainer: this results is consistent with what previously found in other studies [60,61].

Social network and social support have been shown to have a positive impact on health-related quality of life [62,63]. The difficulty with the measurement of this construct lies in the multiplicity of sub-concepts that are part of it and that include social network size, density (i.e. the extent to which the members are connected to each other), homogeneity (i.e. how much individuals are similar to each other in a network) and boundedness (i.e. the degree to which they are defined on the basis of traditional group structures such as kin, work and neighbourhood) [64] and, generally, research focused on only one of these aspects. The COURAGE in Europe SNI, on the contrary, allows for the determination of social networks’ support on the basis of the structural component of the social network of an individual: each component of the network (spouse, parents, other relatives, neighbours, friends and co-workers) provides some kind of support (emotional, instrumental, appraisal and informational), and the frequency of contacts finally provides information on intensity of contacts and on the level of involvement [30]. Therefore, the weighted SNI used in our analysis, by addressing all of the relevant components of social network, enabled us to provide reliable information on the positive effect of a wide and rich social network towards higher QoL.

Finally, our results are completely novel with regard to the positive impact on QoL of living in a house that is perceived as usable and non-risky. Research addressing the impact of built environment features on QoL is almost lacking and, to our knowledge, two previous studies addressed such a relationship. The first refers to a sample of citizens of Bogotá (Colombia) aged 60 or more, in which perceived features of built environment, namely street noise, safety of public areas and street crossings were found to be significantly associated with QoL [65]. In a second study from Italy, the features of built environment were restricted to living in an urban or rural context only, and significant group differences were found only among male respondents [66]. Such a way to address features of built environment is largely partial, as it provides no indication on the degree to which the context in which the persons live has any facilitating or hindering effect. On the contrary our approach, in which different scales measuring perceived environment characteristics were used, enabled us to open to a broader perspective on the association between environmental factors and quality of life: the findings of our study suggest that policies that promote and ensure in-house safety could influence QoL, also controlling for several determinants.

Taken as a whole, our results pointed out similarities and differences in the impact of determinants in the three countries. Some variables–namely smoking status, presence of bodily pain, being emotionally affected by health conditions, as well as having good social network and living in a house that is perceived as usable and with low risk of accidents–had an impact that was basically consistent across countries. For other variables, some differences in their impact across countries were pointed out, in particular connected to age, chronic conditions such as angina and depression, and self-reported feeling of sadness.

Previous studies suggested that age is a predictor of decreased QoL as a result of the effect of chronic conditions [5,34], while in other studies this association was not found [4,35], and our results are in between: if we consider the whole group, the association was confirmed. However, if we take single countries, we see a relevant association for age in Polish population, but not for example among the Spanish, consistently with the results of Garin and colleagues [4]. The same is to be said for angina and depression: they were already found to be associated with and impaired QoL [43,44], but this association was mostly derived from studies performed in clinical populations. In our study, depression was significantly associated to worse QoL only among the Spanish subgroup, thus confirming results previously addressed in other studies where presence of depression was associated to reduced QoL in Spanish populations [67,68]. What it is interesting to note is that among the Polish and Finnish subgroup, feelings of sadness were associated to worse QoL, but not the diagnosis of depression. This remarks the difference between the two variables, and opens to understand the reasons for this trend. We can hypothesize that differences exist in the organization of services in the three countries or that there is higher perception of stigma: a report from Poland seems to point out this element as a relevant one at work, within families and general community members [69].

Some limitations need to be acknowledged. First, the cross-sectional design, that does not allow the investigation of causal relationships. Second, health conditions were self-reported, which could lead to identification bias. Such an approach has been shown to be generally adequate when matched to physicians’ statements on the presence of a disease [70]: anyway, to reduce the risk of reporting biases, generic reference to broad illness group was used, and therefore no definition of disease severity could be applied. Third, data on health state description and on difficulties in daily activities were referred to the previous thirty days, but no indication on the duration of the problem (i.e. how many days in the last thirty?) was included: this might have exerted some effect on variables such as presence of pain, sleep problems or concentration. Finally, we recommend a cautious approach when interpreting the comparison across countries since they should be mostly regarded as a descriptive one. The reason for this is that the three samples are representative of the population of each country, but the way in which respondents were selected, and weights created, was country-dependent: for this reason, no differences across countries were calculated.

In conclusion, we reported information on the determinants of QoL in a sample of community dwelling adults and older adults from Finland, Poland and Spain. Our results shed light on the importance of identifying modifiable risk factors that act as determinants of QoL, and provide public health indications that could support concrete actions at country level. In particular, smoking cessation, increasing the level of physical activity, identifying and addressing pain-related problems, improving social network ties and applying universal design approach to houses and environmental infrastructures could potentially increase QoL of ageing population.

## Supporting Information

S1 File Dataset. (TXT)

S1 Table Full distribution of variables by country. (DOCX)

S2 Table Full Hierarchical regression models to predict quality of life. (DOCX)
