# Supplementary material for: Determinants of Quality of Life in Ageing Populations: Results from a Cross-Sectional Study in Finland, Poland and Spain
Source: PLoS One. 2016 Jul 19;11(7):e0159293. doi: 10.1371/journal.pone.0159293 (PMC4951007; doi:10.1371/journal.pone.0159293)
Supplement: S1 Table — (DOCX) [file pone.0159293.s002.docx]

S1 Table: Full distribution of variables by country

|  | Finland  n=520 | Poland  n=2863 | Spain  n=2256 | Total  n=5639 |
| --- | --- | --- | --- | --- |
| ***Socio/ Demographic*** |  |  |  |  |
| Age in years (mean±sd) | 57.0±16.1 | 44.6±18.3 | 46.1±18.3 | 46.3±18.4 |
| Location |  |  |  |  |
| Urban | 90.3 | 72.3 | 89.4 | 80.6 |
| Rural | 9.7 | 27.7 | 10.6 | 19.4 |
| Sex |  |  |  |  |
| Male | 49.2 | 47.9 | 49.8 | 48.8 |
| Female | 50.8 | 52.1 | 50.2 | 51.2 |
| Education |  |  |  |  |
| None | 0.0 | 0.0 | 2.7 | 1.1 |
| Primary &Secondary | 27.2 | 37.8 | 47.3 | 40.6 |
| High school &Higher education | 72.8 | 62.2 | 50.0 | 58.3 |
| Marital status |  |  |  |  |
| Married/cohabiting | 59.2 | 63.2 | 56.8 | 60.3 |
| Never married | 19.1 | 23.1 | 27.9 | 24.6 |
| Separate/Divorced | 11.7 | 5.5 | 7.4 | 6.8 |
| Widowed | 10.0 | 8.2 | 7.9 | 8.3 |
| ***Health habits*** |  |  |  |  |
| BMI^a^ |  |  |  |  |
| Normal &Underweight | 36.9 | 43.1 | 40.9 | 41.7 |
| Overweight | 39.5 | 33.3 | 37.2 | 35.4 |
| Obese | 23.6 | 23.6 | 21.9 | 22.9 |
| Waist risk |  |  |  |  |
| Low risk | 60.0 | 70.7 | 63.8 | 67.0 |
| High risk | 40.0 | 29.3 | 36.2 | 33.0 |
| Smoking status |  |  |  |  |
| Never smoked | 32.6 | 47.2 | 47.2 | 45.9 |
| Ex-smoker | 48.0 | 21.6 | 18.4 | 22.7 |
| Current smoker | 19.4 | 31.2 | 34.4 | 31.4 |
| Alcohol consumption |  |  |  |  |
| Abstainer | 29.6 | 35.4 | 42.6 | 37.7 |
| Not heavy drinker | 53.4 | 56.4 | 51.8 | 54.4 |
| Infrequent heavy drinker | 15.5 | 7.6 | 4.7 | 7.1 |
| Frequent heavy drinker | 1.5 | 0.6 | 0.9 | 0.8 |
| Physical activity |  |  |  |  |
| Low | 16.3 | 21.7 | 24.2 | 22.2 |
| Moderate | 34.6 | 17.3 | 38.1 | 27.0 |
| High | 49.1 | 61.0 | 37.7 | 50.8 |
| ***Chronic conditions*** |  |  |  |  |
| Arthritis | 37.5 | 14.8 | 11.4 | 15.5 |
| Stroke | 3.4 | 2.0 | 1.7 | 2.0 |
| Angina | 8.1 | 5.0 | 2.9 | 4.4 |
| Diabetes | 8.8 | 6.5 | 6.1 | 6.5 |
| Lung disease | 3.4 | 4.8 | 4.3 | 4.4 |
| Asthma | 9.1 | 5.5 | 7.0 | 6.4 |
| Depression | 15.1 | 7.8 | 15.0 | 11.3 |
| Hypertension | 32.2 | 25.8 | 17.6 | 23.1 |
| ***Health state*** |  |  |  |  |
| Bodily aches or pains |  |  |  |  |
| No pain | 33.1 | 46.5 | 59.5 | 50.4 |
| Pain but no difficulty | 47.2 | 9.1 | 11.1 | 13.3 |
| Pain and mild difficulty  Pain and moderate difficulty | 12.0  6.1 | 19.3  16.9 | 14.9  8.9 | 16.9  12.8 |
| Pain and severe/extreme difficulty | 1.6 | 8.2 | 5.6 | 6.6 |
| Difficulty in learning a new task |  |  |  |  |
| None | 74.6 | 82.5 | 91.0 | 85.2 |
| Mild | 18.9 | 10.3 | 4.7 | 8.8 |
| Moderate | 4.6 | 4.7 | 3.2 | 4.1 |
| Severe/Extreme | 1.9 | 2.5 | 1.1 | 1.9 |
| Difficulty in concentrating |  |  |  |  |
| None | 91.6 | 89.1 | 91.8 | 90.4 |
| Mild | 6.9 | 6.0 | 5.3 | 5.8 |
| Moderate | 0.8 | 3.8 | 2.0 | 2.8 |
| Severe/Extreme | 0.7 | 1.1 | 0.9 | 1.0 |
| Difficulty in sleep |  |  |  |  |
| None | 50.6 | 62.3 | 71.2 | 64.8 |
| Mild | 30.6 | 17.0 | 12.0 | 16.3 |
| Moderate | 10.1 | 12.9 | 10.9 | 11.8 |
| Severe/Extreme | 8.7 | 7.8 | 5.9 | 7.1 |
| Feel tired |  |  |  |  |
| None | 44.1 | 59.2 | 76.7 | 64.7 |
| Mild | 41.1 | 19.4 | 11.8 | 18.4 |
| Moderate | 10.7 | 16.4 | 8.0 | 12.6 |
| Severe/Extreme | 4.1 | 5.0 | 3.5 | 4.3 |
| Feel sad, low or depressed |  |  |  |  |
| None | 72.0 | 50.5 | 71.9 | 60.8 |
| Mild | 21.7 | 27.6 | 15.7 | 22.4 |
| Moderate | 4.8 | 16.7 | 8.5 | 12.4 |
| Severe/Extreme | 1.5 | 5.2 | 3.9 | 4.4 |
| Problems with worry or anxiety |  |  |  |  |
| None | 72.2 | 48.5 | 70.6 | 59.3 |
| Mild | 22.8 | 27.9 | 16.3 | 22.9 |
| Moderate | 3.4 | 18.3 | 9.2 | 13.4 |
| Severe/Extreme | 1.6 | 5.3 | 3.9 | 4.4 |
| Emotionally affect by health problems |  |  |  |  |
| None | 69.8 | 61.5 | 77.3 | 68.5 |
| Mild | 19.5 | 20.5 | 11.6 | 16.9 |
| Moderate | 7.4 | 11.8 | 7.8 | 9.8 |
| Severe/Extreme | 3.3 | 6.2 | 3.3 | 4.8 |
| Difficulties interfere with your life |  |  |  |  |
| None | 64.9 | 60.9 | 77.6 | 67.8 |
| Mild | 24.0 | 20.8 | 11.9 | 17.6 |
| Moderate | 8.4 | 13.7 | 7.4 | 10.8 |
| Severe/Extreme | 2.7 | 4.6 | 3.1 | 3.8 |
| ***Vision/ Hearing*** |  |  |  |  |
| Distant vision |  |  |  |  |
| None | 88.8 | 82.9 | 88.3 | 85.6 |
| Mild | 5.9 | 9.9 | 8.9 | 9.1 |
| Moderate | 3.9 | 5.2 | 2.2 | 3.9 |
| Severe/Extreme | 1.4 | 2.0 | 0.6 | 1.4 |
| Near vision |  |  |  |  |
| None | 89.1 | 82.4 | 88.5 | 85.4 |
| Mild | 7.9 | 10.0 | 9.4 | 9.6 |
| Moderate | 2.2 | 5.9 | 1.7 | 3.9 |
| Severe/Extreme | 0.8 | 1.7 | 0.4 | 1.1 |
| Near hearing |  |  |  |  |
| None | 76.6 | 87.8 | 89.2 | 87.4 |
| Mild | 18.4 | 7.5 | 6.5 | 8.1 |
| Moderate | 4.0 | 3.1 | 3.0 | 3.1 |
| Severe/Extreme | 1.0 | 1.6 | 1.3 | 1.4 |
| Hearing problem in conversation with several people |  |  |  |  |
| None | 87.6 | 86.9 | 91.2 | 88.7 |
| Mild | 10.3 | 8.3 | 5.8 | 7.5 |
| Moderate | 1.7 | 2.7 | 2.3 | 2.4 |
| Severe/Extreme | 0.4 | 2.1 | 0.7 | 1.4 |
| ***SN index*** |  |  |  |  |
| Social Network Score (mean±sd) | 63.0±9.9 | 67.5±12.3 | 74.8±12.5 | 70.0±12.8 |
| ***BE Indexes*** |  |  |  |  |
| Reachability and usability of the neighbourhood environment (mean±sd) | 61.5±20.3 | 69.4±23.4 | 72.9±27.9 | 70.1±25.1 |
| Hinderance of walkable environment (mean±sd) | 14.2±14.9 | 35.2±24.8 | 24.8±26.4 | 29.3±25.5 |
| Open-to-public buildings, places and facilities (mean±sd) | 73.2±18.6 | 65.8±23.8 | 82.8±22.8 | 73.1±24.3 |
| Usability of the living place/home (mean±sd) | 80.6±19.5 | 71.7±23.1 | 82.7±22.7 | 76.8±23.2 |
| ***Quality of Life*** |  |  |  |  |
| Quality of life Score (mean±sd) | 78.1±10.6 | 69.9±14.6 | 74.4±14.9 | 72.4±14.6 |

*Notes.* All values are percentage by country, except where reported differently.  ^a^ Underweight was added to the Normal weight due to low percentages.
