# Supplementary material for: Determinants of Quality of Life in Ageing Populations: Results from a Cross-Sectional Study in Finland, Poland and Spain
Source: PLoS One. 2016 Jul 19;11(7):e0159293. doi: 10.1371/journal.pone.0159293 (PMC4951007; doi:10.1371/journal.pone.0159293)
Supplement: S2 Table — (DOCX) [file pone.0159293.s003.docx]

**S2 Table**: Full Hierarchical regression models to predict quality of life

| **Domains and variables** | **Model 1**  **Socio/ Demographic** | **Model 2**  **Health habits** | **Model 3**  **Chronic conditions** | **Model 4**  **Health state** | **Model 5**  **Vision/ Hearing** | **Model 6 SN Index** | **Model 7**  **BE indexes** | **Model 8**  **Final** |
| --- | --- | --- | --- | --- | --- | --- | --- | --- |
| ***R^2^*** | 0.1793 | 0.2118 | 0.2579 | 0.3725 | 0.3757 | 0.4213 | 0.4539 | 0.4501 |
| ***Socio/ Demographic*** |  |  |  |  |  |  |  |  |
| Country (ref. Poland) |  |  |  |  |  |  |  |  |
| Finland | 10.76*** | 10.06*** | 10.52*** | 7.21*** | 7.09*** | 8.42*** | 7.11*** | 7.40*** |
| Spain | 5.67*** | 6.04*** | 6.24*** | 3.48*** | 3.28*** | 1.30 | -0.06 | 0.34 |
| Location (ref. Urban) | -0.62 |  |  |  |  |  |  |  |
| Age (years) | -0.21*** | -0.20*** | -0.15*** | -0.10*** | -0.09*** | -0.08*** | -0.10*** | -0.09*** |
| Gender (ref. Male) | -1.13 |  |  |  |  |  |  |  |
| Education (ref. Higher education) |  |  |  |  |  |  |  |  |
| None | -7.61*** | -6.70*** | -5.23** | -1.68 | -0.99 | -2.79 | -2.54 | -2.79 |
| Primary + Secondary | -4.87*** | -4.14*** | -3.87*** | -3.10*** | -3.04*** | -2.96*** | -2.43*** | -2.53*** |
| Marital status (ref. Married/cohabiting) |  |  |  |  |  |  |  |  |
| Never married | -1.18 | -1.15 | -1.01 |  |  |  |  |  |
| Separate/Divorced | -5.42*** | -4.78*** | -3.31** |  |  |  |  |  |
| Widowed | -1.62* | -1.42 | -0.57 |  |  |  |  |  |
| ***Health habits*** |  |  |  |  |  |  |  |  |
| BMI (ref. Normal+Underweight) |  |  |  |  |  |  |  |  |
| Overweight |  | 0.37 |  |  |  |  |  |  |
| Obese |  | -0.84 |  |  |  |  |  |  |
| Waist risk (ref. Low risk) |  | -0.83 |  |  |  |  |  |  |
| Smoking status (ref. Never smoked) |  |  |  |  |  |  |  |  |
| Ex-smoker |  | -0.74 | -0.31 | -0.26 | -0.30 | -0.39 | -0.57 | -0.49 |
| Current Smoker |  | -4.01*** | -3.70*** | -3.22*** | -3.18*** | -2.86*** | -2.85*** | -2.80*** |
| Alcohol consumption (ref. Abstainer) |  |  |  |  |  |  |  |  |
| Not heavy drinker |  | 2.88*** | 2.43*** | 1.92** | 1.81** | 1.67** | 1.67** | 1.65** |
| Infrequent heavy drinker |  | 3.17** | 3.35** | 2.57* | 2.58* | 2.40* | 2.46* | 2.46* |
| Frequent heavy drinker |  | 1.98 | 3.81 | 3.10 | 3.01 | 2.62 | 3.66 | 3.66 |
| Physical activity (ref. High) |  |  |  |  |  |  |  |  |
| Low |  | -3.42*** | -3.35*** | -2.47*** | -2.38** | -2.23** | -1.87** | -1.98** |
| Moderate |  | 0.31 | 0.25 | 0.16 | 0.11 | 0.26 | 0.14 | 0.21 |
| ***Chronic conditions*** |  |  |  |  |  |  |  |  |
| Arthritis (ref. No) |  |  | -1.76** | 0.74 |  |  |  |  |
| Stroke (ref. No) |  |  | -1.91 |  |  |  |  |  |
| Angina (ref. No) |  |  | -4.27*** | -2.15* | -2.20* | -2.48** | -2.39** | -2.28* |
| Diabetes (ref. No) |  |  | -1.68* | -0.62 |  |  |  |  |
| Lung disease (ref. No) |  |  | -2.81* | 0.15 |  |  |  |  |
| Asthma (ref. No) |  |  | -1.83 |  |  |  |  |  |
| Depression (ref. No) |  |  | -8.06*** | -3.92*** | -4.01*** | -3.40*** | -3.18*** | -3.18*** |
| Hypertension (ref. No) |  |  | -0.75 |  |  |  |  |  |
| ***Health state*** |  |  |  |  |  |  |  |  |
| Bodily aches or pains (ref. No pain) |  |  |  |  |  |  |  |  |
| Pain but no difficulty |  |  |  | -1.52 | -1.57 | -1.49 | -1.39 | -1.47 |
| Pain and mild difficulty |  |  |  | -2.78** | -2.83** | -3.11*** | -2.70** | -2.70** |
| Pain and moderate difficulty |  |  |  | -2.78** | -2.97** | -3.20*** | -3.31*** | -3.36*** |
| Pain and severe/extreme difficulty |  |  |  | -5.37*** | -5.39*** | -5.85*** | -5.46*** | -5.53*** |
| Difficulty in learning a new task (ref. None) |  |  |  |  |  |  |  |  |
| Mild |  |  |  | 0.25 | 0.35 | -0.46 | -0.24 | -0.34 |
| Moderate |  |  |  | -3.38** | -3.08* | -4.55*** | -4.12*** | -4.21*** |
| Severe/Extreme |  |  |  | -4.34* | -3.75* | -5.95*** | -5.25*** | -5.27*** |
| Difficulty in concentrating (ref. None) |  |  |  |  |  |  |  |  |
| Mild |  |  |  | -2.86* | -2.51* |  |  |  |
| Moderate |  |  |  | -3.17* | -2.68 |  |  |  |
| Severe/Extreme |  |  |  | -5.04* | -4.36 |  |  |  |
| Difficulty in sleep (ref. None) |  |  |  |  |  |  |  |  |
| Mild |  |  |  | -0.87 |  |  |  |  |
| Moderate |  |  |  | -1.80* |  |  |  |  |
| Severe/Extreme |  |  |  | -1.58 |  |  |  |  |
| Feel sad, low or depressed (ref. None) |  |  |  |  |  |  |  |  |
| Mild |  |  |  | -3.23*** | -3.24*** | -2.81*** | -2.85*** | -2.81*** |
| Moderate |  |  |  | -4.40*** | -4.33*** | -3.27*** | -3.58*** | -3.53*** |
| Severe/Extreme |  |  |  | -7.02*** | -7.14*** | -6.38*** | -7.06*** | -7.07*** |
| Emotionally affect by health problems (ref. No) |  |  |  |  |  |  |  |  |
| Mild |  |  |  | -2.78** | -2.89** | -2.90** | -2.58** | -2.66** |
| Moderate |  |  |  | -5.00*** | -5.17*** | -5.30*** | -4.87*** | -4.87*** |
| Severe/Extreme |  |  |  | -4.49** | -4.28* | -4.53** | -4.32* | -4.33* |
| ***Vision/ Hearing*** |  |  |  |  |  |  |  |  |
| Distant vision (ref. None) |  |  |  |  |  |  |  |  |
| Mild |  |  |  |  | -1.25 | -1.69 | -1.10 | -1.20 |
| Moderate |  |  |  |  | -2.61 | -2.87* | -1.66 | -1.77 |
| Severe/Extreme |  |  |  |  | -3.60* | -4.32** | -3.89** | -4.22*** |
| Near vision (ref. None) |  |  |  |  |  |  |  |  |
| Mild |  |  |  |  | -1.24 |  |  |  |
| Moderate |  |  |  |  | -1.20 |  |  |  |
| Severe/Extreme |  |  |  |  | -2.39 |  |  |  |
| Near hearing (ref. None) |  |  |  |  |  |  |  |  |
| Mild |  |  |  |  | -1.04 |  |  |  |
| Moderate |  |  |  |  | -1.29 |  |  |  |
| Severe/Extreme |  |  |  |  | 0.04 |  |  |  |
| ***Social Network index*** |  |  |  |  |  |  |  |  |
| Social Network Score |  |  |  |  |  | 0.28*** | 0.24*** | 0.24*** |
| ***Built Environment Indexes*** |  |  |  |  |  |  |  |  |
| Usability of the neighbourhood environment |  |  |  |  |  |  | 0.01 |  |
| Hindrance of walkable environment |  |  |  |  |  |  | -0.02 |  |
| Easiness of use of public buildings, places and facilities |  |  |  |  |  |  | 0.02 |  |
| Usability of the living place |  |  |  |  |  |  | 0.10*** | 0.11*** |
